# Rod-shaped microglia represent a morphologically distinct subpopulation of disease-associated microglia

**DOI:** 10.1186/s12974-025-03504-5

**Published:** 2025-07-16

**Authors:** Yukio Matsuba, Kenichi Nagata, Yosuke Kadota, Naruhiko Sahara, Takaomi C. Saido, Shoko Hashimoto

**Affiliations:** 1 Pionnering Research Division, Medical Innovation Research Center, Shiga University of Medical Science, Seta Tsukinowa-Cho, Otsu, Shiga 520-2192 Japan; 2 Laboratory for Proteolytic Neuroscience, RIKEN Center for Brain Science, 2-1 Hirosawa, Wako-City, Saitama, 351-0198 Japan; 3 Department of Functional Anatomy and Neuroscience, Nagoya University Graduate School of Medicine, 65 Tsurumai-Cho, Showa-Ku, Nagoya, Aichi 466-8550 Japan; 4 Information Technology and Management Center, Department of Medical Informatics and Biomedical Engineering, Shiga University of Medical Science, Seta Tsukinowa-Cho, Otsu, Shiga 520-2192 Japan; 5 Department of Functional Neurology and Neurosurgery, Brain Research Institute, Niigata University, Chuo-Ku Niigata, Asahimachidori, 951-8585 Japan; 6 Department of Functional Brain Imaging Research, National Institute of Radiological Sciences, National Institutes for Quantum and Radiological Science and Technology, 4-9-1 Anagawa, Inage-Ku, Chiba-City, Chiba, 263-8555 Japan; 7 Department of Neuroscience and Pathobiology, Research Institute of Environmental Medicine, Nagoya University, Furo-Cho, Chikusa-Ku, Nagoya, Aichi 464-8601 Japan

**Keywords:** Rod-shaped microglia, Disease associated microglia (DAM), Oxidative stress, uPA signaling, GAP43

## Abstract

Microglia, resident immune cells of the central nervous system, play an essential role in responding to pathological conditions by adopting diverse activation states and morphologies. Recent single-cell RNA sequencing have revealed that microglial subtypes were heterogeneous based on their gene expression profiles. However, the mechanism on how morphological changes in microglia are correlated with their gene expression profiles remains unclear. The current study aimed to identify a distinct population of rod-shaped microglia, characterized by an elongated morphology, in glutamyl cysteine ligase (GCLC)-deficient mice, a model of glutathione deficiency-induced oxidative stress. In the process of brain atrophy accompanied by neuronal cell death, which was observed in GCLC-KO mice, the rod-shaped microglia emerged in early stages of neurodegeneration and subsequently decreased in number over time. C1q-mediated synaptic pruning has been implicated in microglial activation under pathological conditions. Thus, whether C1q contributes to the formation of rod-shaped microglia was investigated. Notably, the genetic deletion of C1q did not affect the number or distribution of rod-shaped microglia in GCLC-KO mice. These findings suggest that their formation occurs via a C1q-independent mechanism. According to morphological and molecular analyses, the gene expression profile of the rod-shaped microglia was similar to that of the disease-associated microglia (DAM). To investigate the mechanisms underlying their formation, single-nucleus RNA sequencing was performed on cortical tissues collected from GCLC-KO mice. DAM-like microglial clusters were consistently identified. Further, pathway enrichment analysis suggested the potential involvement of the urokinase-type plasminogen activator (uPA, encoded by *Plau*) signaling. Considering the role of uPA in extracellular matrix degradation and cell migration, it may contribute to the morphological changes in rod-shaped microglia. In addition, the phosphorylation of growth-associated protein 43 (GAP43), a modification linked to structural plasticity, increased in rod-shaped microglia. Based on these findings, uPA signaling and phosphorylated GAP43 may be involved in microglial elongation and alignment along neuronal fibers, which potentially facilitate their migration during early neurodegenerative responses. Taken together, the rod-shaped microglia are a previously unrecognized activated population that emerges early in neurodegeneration and may be involved in disease-related processes. Understanding their molecular regulation can provide insights into early microglial responses and potential therapeutic targets.

**Supplementary Information:**

The online version contains supplementary material available at 10.1186/s12974-025-03504-5.

## Introduction

Microglia are responsible for macrophage-like immune responses in the central nervous system. Upon activation, they engage in the phagocytosis of damaged cells, secrete cytokines and chemokines, and undergo dynamic transcriptional and morphological changes. Recent advancements in single-cell RNA sequencing (scRNA-seq) have significantly improved our understanding of microglial biology. Previous studies have revealed that microglia are a highly heterogeneous population with distinct subtypes based on their gene expression profiles [1, 2]. Microglial subpopulations dynamically change during development and disease progression and in response to environmental stimuli [3–5].

Several disease-associated microglial subtypes have been identified in Alzheimer’s disease (AD) mouse models. For example, in the 5XFAD model, two microglial clusters, stage 1 and stage 2 disease-associated microglia (DAM), have been found to be closely associated with the AD pathology [6, 7]. In the *App*^NL−G−F^ knock-in (KI) model, two additional subtypes, activated response microglia (ARM) and interferon response microglia (IRM), have been observed. In particular, ARM and DAM have similar features, and IRM is involved in the interferon signaling [8]. More recently, studies have identified white matter-associated microglia (WAM), which is linked to age-related white matter degeneration, and tau pathology-associated microglia [9, 10]. These findings emphasize the diversity of microglia in neurodegenerative diseases. However, the correlation between microglial morphology and gene expression is still significantly unexplored.

Microglia exhibit distinct morphological changes based on their activation state [11, 12]. Under physiological conditions, they present with small cell bodies with highly ramified processes, which allows for the surveillance of their microenvironment. Upon exposure to inflammatory stimuli or neuronal injury, they transform into an activated state with an enlarged soma and thick, retracted processes. Then, they engage in phagocytosis. In response to severe neurodegenerative conditions, microglia further adopt an amoeboid morphology, characterized by a circular shape with minimal processes, which facilitate enhanced phagocytic activity.

In addition to these well-characterized forms, rod-shaped microglia, a unique microglial morphology, have been described. Rod-shaped microglia, which were first identified during the brain autopsies of patients with syphilitic dementia in 1899 by Franz Nissl (reviewed by Taylor et al.) [13], have since been observed in various neuropathological conditions, including ischemia, dementia with Lewy bodies, and AD [14, 15]. In animal models, rod-shaped microglia have been found in traumatic brain injury models [16, 17], ischemic brain injury [18], and the retina after ocular hypertension [19]. In addition, in vitro studies have shown that they formed in oxygen–glucose deprivation-treated brain slices [20] and in primary microglia cultures after mechanical scratching [21]. The latter study indicated that compared with amoeboid microglia, rod-shaped microglia exhibit a high proliferative capacity and express reduced levels of pro-inflammatory M1 and anti-inflammatory M2 markers [21]. Despite these findings, the molecular identity of rod-shaped microglia in relation to the scRNA-seq-defined microglial subtypes remains unknown. Further, the factors correlated with their formation are still unclear.

Based on a recent report, the neuron-specific conditional knockout of the glutamyl-cysteine ligase catalytic subunit (GCLC) (GCLC^floxed^ X CaMKII-Cre: GCLC-KO) leads to brain atrophy and neuronal loss due to glutathione depletion [22]. Brain atrophy becomes prominent at approximately 4–5 months of age. However, severe neuroinflammation is already evident at 3 months. Notably, a detailed examination of microglial morphology in GCLC-KO mice revealed a diverse range of microglial types, including rod-shaped microglia. This finding indicates that oxidative stress may contribute to the formation of rod-shaped microglia. This study aimed to characterize rod-shaped microglia in GCLC-KO mice via histological analysis and single-nucleus RNA sequencing (snRNA-seq). Our findings can provide novel insights into the molecular identity and potential function of rod-shaped microglia, thereby bridging the gap between microglial morphology and transcriptomic heterogeneity.

## Materials and methods

### Animals

The GCLC^floxed^ mice used in the study were obtained from The European Mouse Mutant Archive. The international strain name for these mice is C57BL/6NTac-Gclctm1a(EUCOMM)Wtsi/WtsiCnbc. The CaMKII-Cre mice were generously provided by Shigeyoshi Itohara from the RIKEN Center for Brain Science. The GCLC^floxed^ mice crossed with CaMKII-Cre mice were referred to as GCLC-KO for convenience. GCLC protein levels were markedly reduced in the cortex of GCLC-KO mice, as confirmed by Western blotting (Supplemental Fig. 1A). C1qa-deleted mice were generated by CRISPR/Cas9-mediated genome editing. The mice were housed in a controlled environment maintained at 22 °C with a 12-h light/dark cycle. The bedding material was provided in individual cages. Food and water were available ad libitum. The personnel involved in handling wore appropriate personal protective equipment, including gloves and lab coats. The mice were gently handled and acclimated to human contact to decrease stress.

### Immunohistochemistry

Immunostaining was conducted using fixed frozen Sects. (20-μm thick) of various brain samples or vibratome Sects. (50- or 100-μm thick). For the frozen sections, antigen retrieval was performed by autoclaving at 121 °C for 5 min in 10mM sodium citrate buffer (pH 6.0) based on the antibody used to activate the antigen. Thereafter, endogenous peroxidase was inactivated by 0.3% hydrogen peroxide in methanol. To block nonspecific immunoreactivity, the sections were treated with the blocking solutions (0.2% Casein in phosphate buffered saline [PBS]). Primary antibodies (Supplemental Table 1), diluted in Tris-HCl and NaCl (TN) buffer (0.1 M Tris-HCl, 0.15 M NaCl, pH 7.5), were reacted overnight at 4 °C. The sections were then washed three times in Tris-HCl, NaCl, and Triton (TNT) buffer (TN buffer containing 0.05% TritoX-100) for 5 min. Then, the sections were treated with Alexa 488- or Alexa 555-conjugated anti-mouse/rabbit IgG (1:500 dilution, Molecular Probes, Eugene, OR). Finally, the sections were cover-slipped using ProLong Gold Antifade Mountant (Thermo Fisher Scientific, San Jose, CA, the USA). For the phosphorylation of growth-associated protein 43 (pGAP43) staining, signal amplification was performed using the TSA kit. Meanwhile, for C1q staining, antigen retrieval was performed using protease treatment. For the vibratome sections, after blocking with blocking solution (0.2% casein, 0.3% Triton X-100, and 3% goat serum diluted in PBS) for 2 h at room temperature, the primary antibody was reacted overnight at 4 °C. The sections were then washed with PBS and treated with secondary antibody (diluted in PBS containing 0.3% Triton X-100) for 2 h at room temperature. After washing with PBS, the sections were cover-slipped. The immunostained sections were scanned on the NanoZoomer NDP system (Hamamatsu Photonics, Shizuoka, Japan) with 20 × resolution, FLUOVIEW FV3000 (Evident, Tokyo, Japan), or TCS SP8 X (Leica, Wetzlar, Germany).

### In situ hybridization using RNAscope

The probes used for each gene in the RNAscope analysis were purchased from Advanced Cell Diagnostics (Advanced Cell Diagnostics, CA, the USA). Supplemental Table 2 shows the product numbers. The in situ hybridization by RNAscope was conducted based on the protocol requiring RNAscope 2.5 HD Assay-Red (Advanced Cell Diagnostics). After the in situ hybridization, immunostaining was conducted using the Iba1 antibody, as described in the Immunohistochemistry section.

### Morphological classification criteria

Microglial subtypes were classified based on Iba1 immunostaining using quantitative morphological criteria. First, rod-shaped microglia were defined as Iba1-positive structures with a total length exceeding 50 μm. Among the remaining Iba1-positive cells, those with a cell body area greater than 100 μm^2^ were classified as amoeboid microglia. Cells that did not meet these criteria and exhibited branched processes were classified as ramified microglia. These criteria were applied consistently across all images used for analysis.

For the graphs shown in Figs. 4 and 6, two types of quantification were performed: First, the yellow bars, representing the ratio of target RNA-positive area to Iba1-positive area, were calculated from confocal microscopy images obtained by Iba1 immunohistochemistry combined with RNAScope in situ hybridization. Rather than using entire image fields, quantification was performed at the level of individual Iba1-positive microglial structures. For each structure, the ratio of target RNA-positive area to Iba1-positive area was calculated. Morphology-enriched regions (rod-, amoeboid-, and ramified-rich areas) were manually identified—two rod-rich areas and one each for amoeboid and ramified per animal. The number of microglial structures analyzed per morphology per animal ranged from approximately 7 to 60, depending on the confocal field. Exact cell counts for each figure and condition are summarized in Supplemental Table S3. Image analysis was conducted using Hybrid Cell Count BZ-H3C software (Keyence, Osaka, Japan). Second, the orange bars, representing the ratio of target RNA-positive cells to Iba1-positive cells, were calculated using whole-section images acquired with a NanoZoomer scanner. Morphology-enriched areas were manually annotated (Supplemental Fig. 3A), and for each morphology, at least 40 Iba1-positive microglial structures were randomly selected and analyzed per animal. If fewer than 40 structures were available (e.g., in rod-rich areas), all observable ones were included. For Fig. 6, orange bar data were calculated using confocal images, and the same quantification procedure was applied as described above. As the orange bar data in Fig. 6 were derived from confocal images, the number of analyzable microglial structures was often below 40; in such cases, all observable structures were analyzed per animal. Comprehensive details regarding animal numbers, imaging methods, brain regions analyzed, cell counts, and image selection criteria are summarized in Supplemental Table S3.

### Tissue clearing using AbScale

The transparency processing of mouse brain tissues was conducted based on the method described by Hama et al. [23, 24] and the attached protocol of SCALEVIEW-S (Fujifilm, Tokyo, Japan). The reagents used for the AbScale were purchased from Fujifilm and kindly provided by Dr. Hama. The brain of a 3-month-old GCLC-KO mouse was extracted, and 1mm-thick coronal brain slices were prepared. The brain slices were incubated in ScaleS0 for 8 h, followed by ScaleA2 for 8 h, 8 M Urea for 8 h, and finally ScaleA2 for 12 h at 37 °C. After incubating in DeScale Solution for 6 h at 4 °C, the slices underwent primary antibody reaction (anti-Iba1 antibody, 1:500) at 37 °C for 90 h. Subsequently, they were treated with AbScale for 2 h at room temperature, followed by incubation with AbScale for 1 more hour at room temperature. Thereafter, secondary antibody reaction (Donkey anti-rabbit Alexa PLUS 488, 1:500) was conducted at 37 °C for 48 h, followed by incubation with AbScale for 2 h at room temperature and then incubation with AbScale for 1 more hour at room temperature. Finally, the slices were fixed in 4% PFA for 40 min, washed with PBS, and incubated in ScaleS4 at 37 °C for 8 h. The observation of stained transparent tissues was performed using the FV3000 inverted fluorescence microscope.

### Hematoxylin and eosin staining

Hematoxylin and eosin staining was performed as follows: After deparaffinization, the tissue sections were stained with Mayer’s hematoxylin solution (Wako, Tokyo, Japan) for 10 min, followed by staining with eosin alcohol solution (Wako) for 4 min. The sections were then dehydrated via an ethanol series, cleared, and cover-slipped using the NEW MX mounting medium (Matsunami Glass Ind., Ltd.).

### Western blot analysis

The frozen cortex was homogenized in a homogenize buffer (50 mM Tris-HCl, 150 mM NaCl [pH 7.5] containing 1% Triton X-100, and a protease inhibitor cocktail). The samples were subjected to ultracentrifugation at 20,000 x g for 15 min at 4 °C. The supernatants were collected and used for western blot analysis. Protein concentrations were determined using the BCA protein assay kit (Pierce, Rockford, IL, the USA). An equivalent amount of protein was mixed with a 4X sample buffer with 2-mercaptoethanol. The protein samples were separated via sodium dodecyl-sulfate-polyacrylamide gel electrophoresis and transferred to a PVDF membrane (Merck Millipore, Burlington, MA, the USA) via electrophoretic transfer. The membrane was treated with ECL™ prime blocking solutions (Cytiva, Tokyo, Japan) and reacted overnight at 4 °C with each antibody (Supplemental Table 1) diluted in blocking buffer. The membrane was then washed three times with Tris-buffered saline containing Tween 20 (TBS-T) for 10 min. Then, it was incubated with HRP-conjugated anti-rabbit or anti-mouse IgG (Cytiva) for 1 h. The immunoreactive bands on the membrane were visualized using ECL Select (Cytiva) and scanned with the LAS-4000mini LuminoImage analyzer (Fuji Film) or LuminoGraph I (Atto, Tokyo, Japan). The intensity of each band was quantified using Image Studio™ Software (LI-COR Biosciences, Lincoln, NE, the USA).

### Single nuclei RNA sequencing

The regions of the cerebral cortex from 3- and 6-month-old GCLC-KO mice with abundant rod-shaped microglia were utilized. The brain samples were homogenized in 2 mL of Nuclei EZ Lysis Buffer (Merck Millipore) and centrifuged at 500 × g for 5 min at 4 °C. After centrifugation, the pellet was resuspended in EZ Lysis Buffer and centrifuged again at 500 × g for 5 min at 4 °C. The supernatant was removed, and the pellet was resuspended in ice-cold PBS (containing 0.04% BSA and 40 U/mL RNase inhibitor). The nuclear suspension was stained with 4’,6-diamidino-2-phenylindole and passed through the FlowMi cell strainer. Cell sorting via fluorescence-activated cell sorting was performed to remove debris and obtain fractions containing only single nuclei. The libraries for scRNA-seq were prepared using the 10X Genomics Chromium Single-Cell 3’ Reagent kits (version 3, Pleasanton, CA, the USA) based on a standardized procedure. Then, the amplified cDNA was used to construct a barcoded 3′ library according to the manufacturer’s instructions. Paired-end sequencing was performed on Illumina NovaSeq 6000 (San Diego, CA, the USA). The library preparation was conducted by the Research Resource Division (RRD) of RIKEN Center for Brain Science. Sequencing was conducted by Azenta Life Sciences (Tokyo, Japan).

### Data analysis of snRNA-seq

Raw FASTQ files were processed to generate barcodes.tsv, features.tsv, and matrix.mtx files by AMELIEF Co., Ltd. (Tokyo, Japan), using Cell Ranger pipelines. Sequence data were analyzed using Seurat version 5.1.0 [25] implemented in R version 4.3.2. First, count matrices were imported into Seurat using the Read10X function, and Seurat objects were created for each sample with the CreateSeuratObject function. Quality control was performed by excluding nuclei with > 7,000 detected genes or mitochondrial gene content > 1%, resulting in a final dataset of 30,516 nuclei. Data normalization and variance stabilization were conducted using the SCTransform function, which also identified highly variable genes and applied data scaling. Dimensionality reduction was performed via principal component analysis with RunPCA, followed by clustering with FindNeighbors and FindClusters, with a resolution of 0.2. For visualization, Uniform Manifold Approximation and Projection (UMAP) was conducted based on the top 30 principal components using the RunUMAP function. Data from each sequence were integrated with the IntegrateLayers function. Additional clustering analyses of *Hexb* + microglia were then performed. Microglia clusters expressing other cell-type markers, such as *St18* (for oligodendrocytes) and *Rbfox3* (for neurons), were considered as doublets and eliminated from the subsequent analyses. The *Mrc1* + macrophage cluster was also eliminated as non-microglia cluster. The marker genes for each subcluster of microglia were searched using the FindAllMarkers function (logfc.threshold = 1, min.pct = 0.05), with the default Wilcoxon rank-sum test. Pathway enrichment analysis was conducted using Enrichr (https://maayanlab.cloud/Enrichr/) with the Reactome 2024 pathway database. Significantly enriched biological pathways were determined based on the adjusted *p*-values and combined scores provided by Enrichr. Figure 5E shows the top pathways, and Supplemental Data 3 depicts the complete dataset.

### Re-analysis of public scRNA-seq datasets using Seurat

The public scRNA-seq datasets from 5XFAD (GSE140510) and *App*^NL−G−F^ KI mice (GSE127893) were reanalyzed according to a previous study [10]. FeaturePlot and VlnPlot were used to visualize *Plau* and DAM marker expression across conditions (WT, TREM2-KO, 5XFAD, TREM2-KO X 5XFAD, and various ages in *App*^NL−G−F^). Statistical comparisons of gene expression levels across genotypes (e.g., WT, 5XFAD, TREM2-KO, and TREM2-KO X 5XFAD) and across age groups (in *App*^NL−G−F^ mice) were performed using the Wilcoxon rank sum test. When multiple comparisons were conducted, Bonferroni correction was applied to adjust the *p*-values. The statistical results for selected genes are summarized in Supplemental Data 4.

### Generation of C1qa-deleted mice

The *C1qa*-deleted mice were generated using the CRISPR/Cas9 gene editing technology. The *C1qa* gene specific short guide RNA (sgRNA) was designed in silico using CHOPCHOP (https://chopchop.cbu.uib.no/), an online tool. Template oligo DNAs were designed to create two sgRNAs by annealing the sense and antisense strands of the sequences “CTGCCGAGCACCCAACGGGAAGG” and “GTAAGCGTTCTCTCCGGCTGGGG”. The sgRNA was transcribed using the MEGAshortscript T7 Transcription Kit (Thermo Fisher Scientific) with generated templates. Subsequently, the sgRNAs were purified with the MEGAClear Transcription Clean-Up Kit (Thermo Fisher Scientific). The Cas9 mRNA and sgRNA were injected into the GCLC-KO mouse zygotes using the micro-injection system. The zygotes were incubated at 37 °C in 5% CO2 until two-cell embryos had developed, which were then transferred to the recipient mice. The micro-injection and mouse breeding procedures were provided by the Support Unit for Animal Resource Development, RRD of RIKEN Center for Brain Science (CBS). Genomic DNA was extracted from the tails and brain tissues using the standard ethanol precipitation method. The DNA fragments for sequencing were obtained via polymerase chain reaction (PCR) using the following primers: forward primer 5′-CCTTGCGTGTAGAACATGGTAA-3′, reverse primer 5′-ATCGGAAGAGAACCTGGAAACA-3′. Genomic DNA was extracted with the KOD FX Neo DNA polymerase (Toyobo, Osaka, Japan). Sanger sequencing was performed using the forward primer and the PCR product with the BigDye Terminator v3.1 Cycle Sequencing kit (Thermo Fisher Scientific). As a result of genome editing, bases 83–164 of the coding sequence were deleted, resulting in the loss of the C1q protein expression.

### RNA isolation and quantitative real-time PCR

Total RNA was extracted from cortical tissue samples using RNAiso Plus (Takara, Shiga, Japan), according to the manufacturer’s instructions. Briefly, tissues were homogenized in 500 μL of the RNAiso Plus reagent, followed by the addition of 100 μL of chloroform. After thorough mixing and centrifugation, the aqueous phase was collected, and the total RNA was precipitated with isopropanol. The RNA pellet was washed and dissolved according to the standard protocol. Genomic DNA was removed, and reverse transcription was performed using the ReverTra Ace qPCR RT Kit (Toyobo, Osaka, Japan), according to the manufacturer’s instructions. Quantitative real-time PCR (qRT-PCR) was carried out using the QuantStudio 12 K Flex Real-Time PCR System (Thermo Fisher Scientific) with THUNDERBIRD™ SYBR® qPCR Mix (Toyobo). Each reaction contained 6 pmol of gene-specific primers and aliquots of synthesized cDNA. The PCR cycling conditions were as follows: 50 °C for 2 min, 95 °C for 1 min, followed by 40 cycles of 95 °C for 10 s and 60 °C for 30 s. All qRT-PCR assays were performed in technical duplicates. The relative mRNA expression levels were calculated using the standard curve method. The gene expression levels were normalized to the Gapdh expression as an internal control. The primer sequences used in this study were described in our previous report [22].

### Statistical analysis

All analyses were performed using the GraphPad Prism 10 software (San Diego, CA, the USA). The Student’s *t*-test was used to assess statistically significant differences.

## Results

### Rod-shaped microglia that emerged before neurodegeneration became prominent in oxidative stress-enhanced mice

**Fig. 1:**
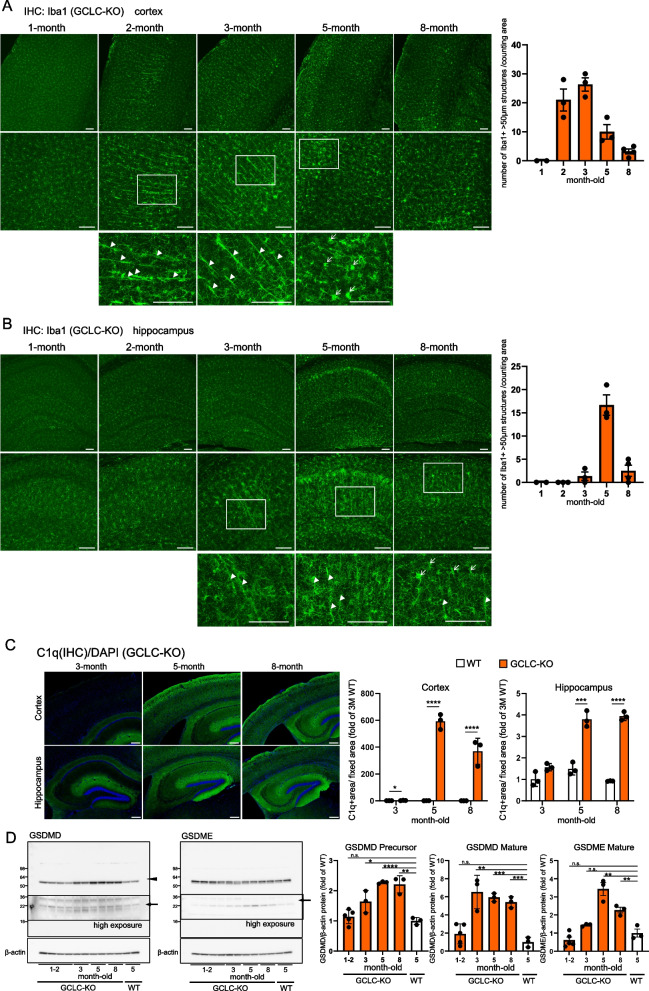
Rod-shaped microglia emerge prior to overt neurodegeneration. **A**, **B** Brain sections from 1- to 8-month-old GCLC-KO mice were immunostained with an anti-Iba1 antibody. **A** shows the cortical region, and **B** shows the hippocampal region. In both **A** and **B**, the top row presents wide-field images acquired using 10 × confocal microscopy. The second row shows higher-magnification (20 ×) images of selected time points (2, 3, and 5 months for cortex; 3, 5, and 8 months for hippocampus). The third row provides enlarged views of the areas outlined by rectangles in the second row. In the third-row images, rod-shaped microglia (length ≥ 50 μm) are marked with arrowheads, and amoeboid microglia (area ≥ 100 μm^2^) are marked with arrows. The graph presents the number of rod-shaped (Iba1-positive) structures ≥ 50 μm in length counted within the 10 × confocal image fields. Values from 2 to 8 months are expressed as mean ± SEM (*n* = 3 or 4 mice). Scale bar: 100 μm (applies to all images). **C** The brain sections from 3- to 8-month-old GCLC-KO mice were immunostained with the C1q antibody. The upper panels show the cortical region, and the lower panels depict the hippocampal region. The values shown in the graph are the fluorescence intensity of C1q in wild-type (WT) and GCLC-KO mice with the results expressed as the mean relative levels ± SEM (*n* = 3, n.s.: not significant). Supplemental Fig. 2A shows the immunostaining images of the wild-type mice. The scale bar represents 200 μm. *p < 0.05, *** *p* < 0.001, **** p < 0.0001. **D** Western blot analysis of the GSDMD and GSDME protein levels in the cortex of GCLC-KO mice at different ages. Representative blots of the GSDMD precursor, GSDMD mature, and GSDME mature are shown, with β-actin as the loading control. The quantification of protein levels was normalized to β-actin and expressed as fold change relative to wild-type controls. The data were presented as the mean ± SEM (*n* = 3 or 5, *p < 0.05, **p < 0.01, *** p < 0.001, **** p < 0.0001; n.s.: not significant)

To investigate the impact of oxidative stress on microglial morphology, brain slices immunostained with the Iba1 antibody were examined. Notably, the glutathione-deficient (GCLC-KO) mice, but not the wild-type mice, presented with the rod-shaped microglia with an elongated morphology (Fig. 1A, B and Supplemental Fig. 1B). The temporal dynamics of the rod-shaped microglia in GCLC-KO mice was initially analyzed. Their numbers peaked at 2–3 months in the cerebral cortex and at 5 months in the hippocampus and then gradually declined (Fig. 1A and B). This finding is contrast to that in severe neuroinflammation, with numbers peaking at 4–5 months in GCLC-KO mice [22], and amoeboid microglia, with numbers peaking at 5 months in the cortex and 5–8 months in the hippocampus (Supplemental Fig. 1C). Based on these details, the rod-shaped microglia emerge earlier than the amoeboid microglia. Spatially, amoeboid microglia were often observed in the cortical layers II/III, which are among the regions where neurodegeneration appeared most prominent. Meanwhile, rod-shaped microglia extended from layer V toward the outer cortical regions, with cells aligned in the same direction. In the hippocampus, both the amoeboid and rod-shaped microglia were concentrated in CA1, a region severely affected by neurodegeneration. The rod-shaped microglia in CA1 extended from the pyramidal cell layer toward the dentate gyrus.

The GCLC-KO mice exhibit significant brain atrophy and neuronal cell death starting at approximately 5 months of age [22]. Based on a previous report, C1q accumulation at the synapses, marking microglia-mediated synaptic phagocytosis, and gasdermin D/E (GSDMD, GSDME)-mediated pyroptosis contribute to neuronal death in GCLC-KO mice [22]. Gasdermins facilitate cytokine release and cell death via pore formation. Thus, the temporal association between rod-shaped microglia, C1q activation, and gasdermin-mediated pyroptosis was investigated (Fig. 1C and D). The rod-shaped microglia peaked at 2–3 months in the cortex. However, immunohistochemical analysis revealed that C1q activation peaked at 5 months and remained elevated until at least 8 months (Fig. 1C, Supplemental Fig. 2A). Similarly, western blot analysis showed that gasdermin activation occurred after the decline of the rod-shaped microglia (Fig. 1D). Therefore, rod-shaped microglia emerge prior to overt neurodegeneration, suggesting they may be part of an early microglial response to neuronal stress.

### Rod-shaped microglia formed elongated shapes by connecting multiple cells

**Fig. 2:**
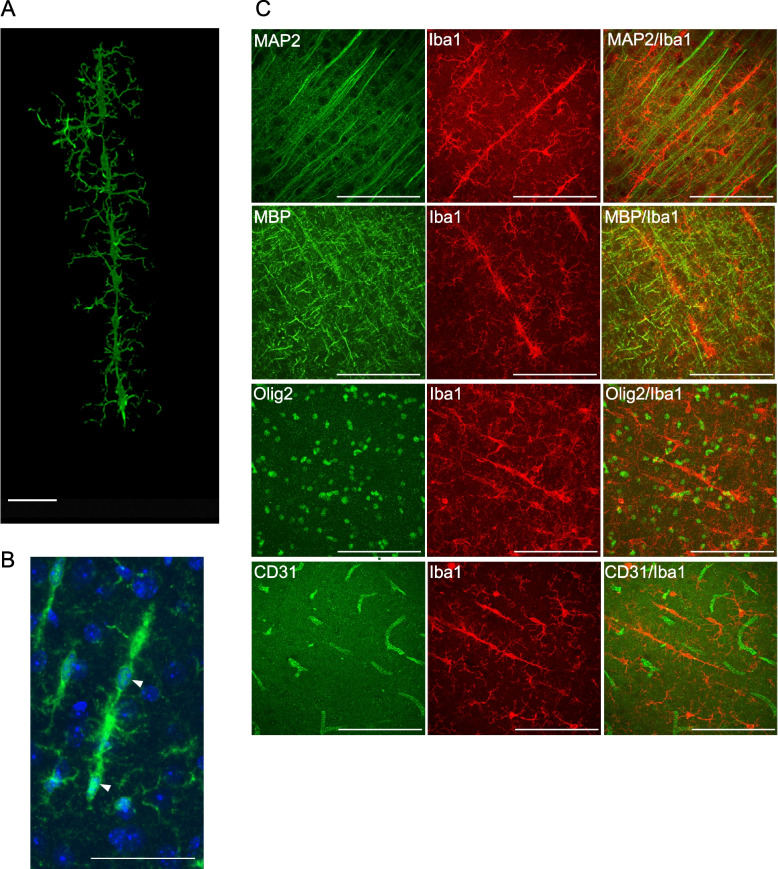
Rod-shaped microglia elongated the along nerve fibers. **A** After transparentizing 1mm thick slices using a Sca*l*e, immunostaining was conducted using the Iba1 antibody. Images were captured using a confocal microscope and reconstructed into three-dimensional images after Z-stack. The signals from the surrounding microglia were erased to focus solely on single rod-shaped microglia. The scale bar represents 50 μm. Supplemental Data 1 depicts the video file. **B** The brain sections from 3-month-old GCLC-KO mice were immunostained with the Iba1 antibody and stained with Hoechst. The scale bar represents 100 μm. **C** The brain sections from 3-month-old GCLC-KO mice were immunostained with the MAP2 (dendrite marker)/MBP (myelin marker)/Olig2 (oligodendrocyte marker)/CD31 (endothelial cell marker), and the Iba1 antibodies. The scale bar represents 100 μm

To obtain a comprehensive understanding of the structural characteristics of rod-shaped microglia, staining on a 1mm-thick cleared brain tissues was conducted using the Iba1 antibody with the AbSca*l*e technique (Fig. 2A). Subsequently, three-dimensional images were acquired using a confocal microscope. Supplemental Data 1 presents a three-dimensional reconstruction of these rod-shaped microglia. Results revealed that the cell bodies of the microglia exhibit swelling and elongation in the rod-shaped microglia, accompanied by several neurites. In addition, in accordance with the findings of several researchers [19, 20], some cells were evidently interconnected, forming elongated structures (Fig. 2B).

Further, the correlation between the rod-shaped microglia and surrounding structures was investigated. Results showed that the rod-shaped microglia extended along the dendrites stained with the MAP2 antibody and the myelin stained with the MBP antibody (Fig. 2C). This finding was also reported by Ziebell et al. [17]. Nevertheless, no positive correlation was observed between the rod-shaped microglia and other structures including oligodendrocytes, and endothelial cells (Fig. 2C). Based on these results, we hypothesized that rod-shaped microglia communicate with neuronal cells and other microglial cells via their neurites in the early stages of neurodegeneration.

### C1q deficiency did not affect the formation of rod-shaped microglia

The rod-shaped microglia exhibit an elongated morphology, and they are aligned along neuronal fibers, which suggest that they interact closely with neuronal structures. This raises the possibility that their formation is induced by signals originating from neurons undergoing stress or degeneration. As shown in Fig. 1C and Supplemental Fig. 2A, C1q expression was elevated in the cortex at least by 3 months of age in GCLC-KO mice compared with wild-type controls. It is possible that rod-shaped microglia respond to early neuronal signals. Although C1q expression appears to rise slightly after the initial emergence of rod-shaped microglia in the cortex, it may contribute to the formation or maintenance of this morphology under oxidative stress conditions. C1q plays a key role in synaptic pruning by tagging synapses for microglial phagocytosis. Under normal conditions, this process contributes to the refinement of neural circuits [26, 27]. However, under pathological conditions such as AD, excessive C1q deposition leads to aberrant synapse loss, which is associated with cognitive decline [27]. This indicates that dysregulated C1q-mediated synaptic pruning contributes to neurodegeneration by enhancing microglial synaptic engulfment beyond physiological levels.

**Fig. 3:**
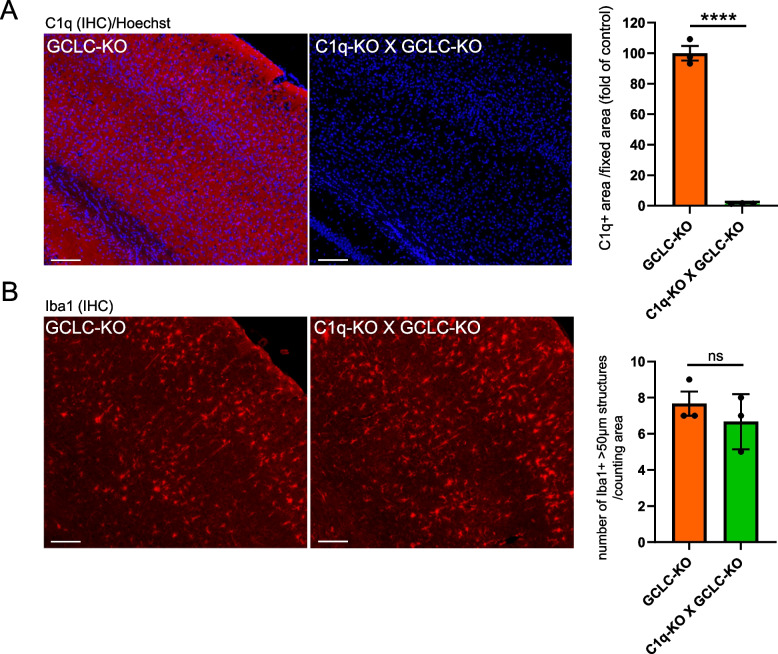
C1q deficiency did not affect the formation of rod-shaped microglia. **A** Immunohistochemistry for C1q in the brain sections of GCLC-KO and C1q-KO X GCLC-KO mice. The cortical region is shown. The graph on the right represents the quantification of C1q immunoreactivity (expressed as fold change relative to GCLC-KO). Data were presented as the mean ± standard error of the mean (*n* = 3). ****p < 0.0001. The scale bar represents 100 μm. **B** Immunohistochemistry for Iba1 in the brain sections of GCLC-KO and C1q-KO X GCLC-KO mice showing rod-shaped microglia. The graph on the right depicts the number of rod-shaped microglia per defined area in both genotypes. Data were expressed as the mean ± standard error of the mean (*n* = 3). n.s., not significant. The scale bar represents 100 μm

Therefore, we hypothesized that C1q knockout may influence the morphological transition to rod-shaped microglia by suppressing abnormal synapse detection by the microglia. To test this notion, C1q knockout mice (C1q-KO) were generated and crossed with GCLC-KO mice to establish C1q-KO X GCLC-KO double-mutant mice (Fig. 3A, Supplemental Fig. 2C). To determine the contribution of C1q to the formation of the rod-shaped microglia, the C1q mRNA expression in the microglia with different morphologies was initially analyzed. The C1q expression levels among the ramified, amoeboid, and rod-shaped microglia were comparable (Supplemental Fig. 2B). Next, whether genetic deletion of C1q affected the formation of rod-shaped microglia was examined. The number and distribution of rod-shaped microglia in C1q-KO X GCLC-KO double-mutant mice were similar to those in GCLC-KO mice (Fig. 3B). Of note, both brain volume (Supplemental Fig. 2D) and the expression of DAM and other microglial activation markers (Supplemental Fig. 2E) were comparable between GCLC-KO and C1q-KO X GCLC-KO mice, even though these markers were significantly altered in GCLC-KO mice compared with wild-type controls based on a previous study [22]. Taken together, these results indicate that the formation of rod-shaped microglia is not dependent on C1q-mediated synaptic tagging or complement activation. Rather, rod-shaped microglia are likely to be induced by alternative pathways, independent of the complement cascade.

### Rod-shaped microglia predominantly expressed DAM markers

The formation of rod-shaped microglia appears to be independent of C1q-mediated synapse tagging. Thus, the gene expression profile of rod-shaped microglia observed in GCLC-KO mice was analyzed next. Several microglial subpopulations have been identified and reported via scRNA-seq analyses utilizing various mouse models. For example, in the 5XFAD AD mouse model, two distinct clusters of DAM have been characterized: stage 1 and stage 2 DAM clusters, which are closely associated with neurodegenerative conditions [7]. According to previous studies, the formation of stage 2 DAM is driven by signal transduction via Trem2 [6, 7]. Moreover, in the *App*^NL−G−F^ KI mouse model, two distinct microglial subpopulations have been identified. The first is the ARM, which exhibits characteristics reminiscent of DAM, and the second is the IRM, which plays an essential role in the interferon response [8]. These findings emphasize the diversity of microglial subpopulations and their specific roles, thereby shedding light on the complex and dynamic nature of microglial responses.

**Fig. 4:**
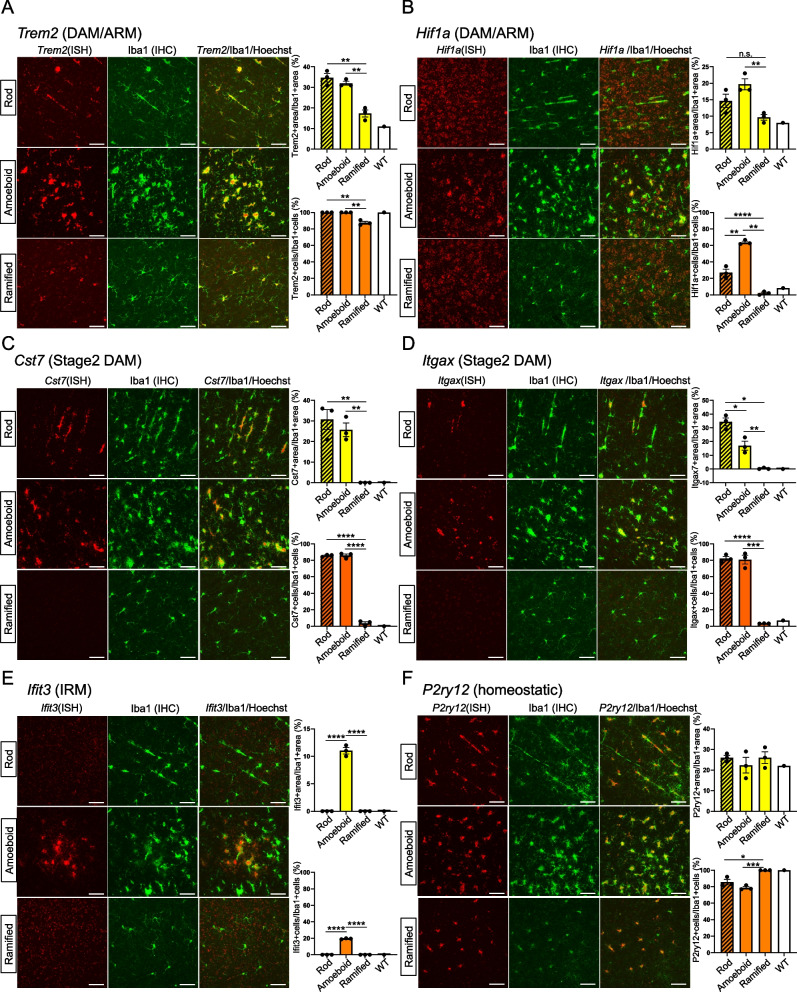
Rod-shaped microglia expressed DAM markers. **A**–**E** The brain sections from 3-month-old GCLC-KO mice were subjected to dual staining using in situ hybridization with RNAscope and immunostaining with the Iba1 antibody. Each panel depicts the regions with abundant rod, amoeboid, and ramified microglia (Supplemental Fig. 3A). The scale bar represents 50 μm. The upper graph represents the ratio of the RNAscope-positive area to the Iba1-positive area. Meanwhile, the lower graph indicates the ratio of RNAscope-positive cells to Iba1-positive cells. Data for rod-, amoeboid-, and ramified-type microglia in GCLC-KO mice are presented as mean ± standard error of the mean, based on three biological replicates (*n* = 3 mice). The reference values for microglia in wild-type mice (white bar) were also provided. Supplemental Fig. 3B presents the staining images of wild-type mice. *p < 0.05, **p < 0.01, *** p < 0.001, **** p < 0.0001

To investigate the characteristics of rod-shaped microglia, the expression patterns of previously reported microglial cluster marker genes in GCLC-KO mice were examined. This was accomplished by double staining, involving immunohistochemistry for Iba1 and RNAscope for marker genes. The rod-shaped and amoeboid microglia in the cerebral cortex region of GCLC-KO mice and the ramified microglia in the midbrain region of the same brain sections were examined. This is because microglial activation is minimal in the midbrain and most microglia remain in a ramified state (Supplemental Fig. 3A). As DAM markers, the expression patterns of *Cst7*, *Itgax*, *Trem2*, and *Hif1a* in rod-shaped, amoeboid, and ramified microglia were investigated (Fig. 4A–D, Supplemental Fig. 3B, C). Using the area and the ratio of length to width of each cell, the cell morphology was classified into ramified, amoeboid, and rod, and the expression levels of markers for each morphology was quantified. Further, the RNAscope signals were quantified in two ways: (1) the relative expression level per microglia, represented by the ratio of the RNAscope-positive area to the Iba1-positive area (each upper graph), and (2) the proportion of microglia expressing the target gene, represented by the ratio of RNAscope-positive cells to Iba1-positive cells (each lower graph). *Trem2* is expressed in most microglia, including those in wild-type mice. However, its expression levels are believed to be stronger in amoeboid and rod-shaped microglia (Fig. 4A). For *Hif1a*, the number of positive cells significantly increased in rod-shaped microglia (Fig. 4B). As for *Cst7* and *Itgax*, which are prominently expressed in stage 2 DAM, they were mostly negative in the ramified microglia. However, in the rod-shaped microglia—similar to the amoeboid microglia—the number of positive cells was significantly higher (Fig. 4C and D). Further, *Ifit3*, an IRM marker, was expressed in a portion of the amoeboid microglia but not in the rod-shaped microglia (Fig. 4F). The levels of *P2ry12*, which is a marker of homeostatic microglia, decrease in DAM. Indeed, in GCLC-KO mice, a reduction in *P2ry12* levels was observed in some amoeboid microglia, both at the mRNA and protein levels (Fig. 4F and Supplemental Fig. 3C). The rod-shaped microglia also exhibited reduced *P2ry12* levels (Fig. 4F). At the protein level, both the P2ry12-positive and P2ry12-negative cells were observed (Supplemental Fig. 3D). Based on these results, rod-shaped microglia predominantly express DAM-associated genes. These results suggest that rod-shaped microglia share DAM-associated gene expression features and exhibit reduced P2ry12 expression, similar to amoeboid microglia.

### snRNA-seq revealed *Plau* enrichment in DAM-like rod-shaped microglia

**Fig. 5:**
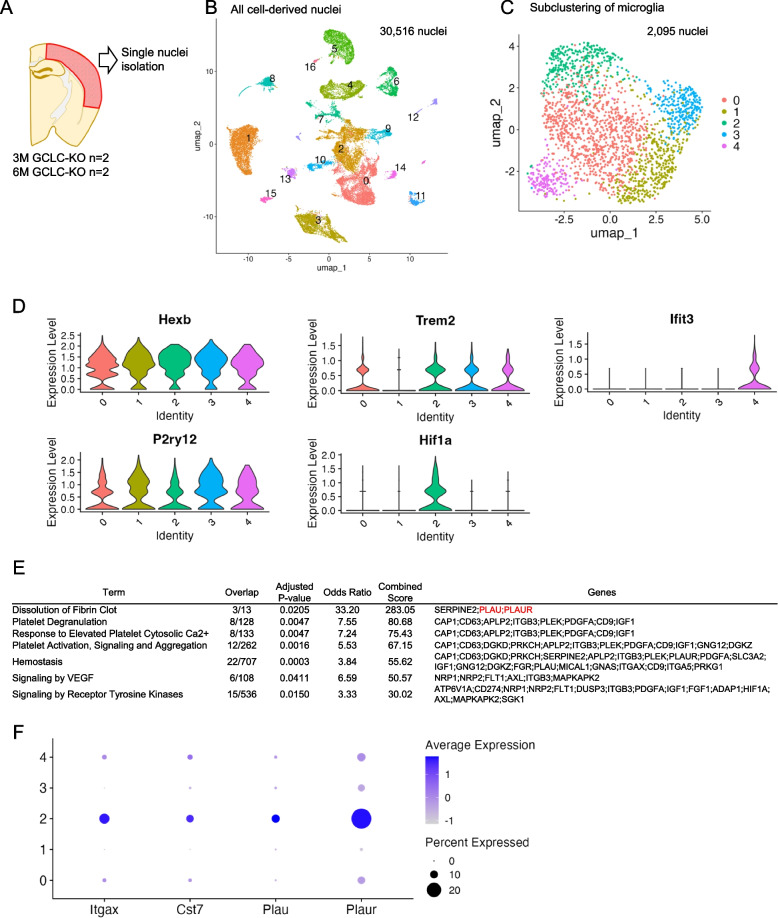
Gene expression profiling of microglia in GCLC-KO mice. **A** The tissue from the cortical regions with a high abundance of rod-shaped microglia (indicated in red) was dissected, and single nuclei were isolated. **B** The dataset included samples from 3-month-old (*n* = 2) and 6-month-old (*n* = 2) GCLC-KO mice, which were integrated for analysis. The uniform manifold approximation and projection (UMAP) plot represents the clustering of nuclei based on transcriptomic profiles, thereby emphasizing distinct cell populations present in these regions. **C** The microglia clusters were extracted from the integrated snRNA-seq dataset and further subjected to subclustering analysis. The resulting subclusters were visualized using UMAP. The dataset included samples from 3-month-old (*n* = 2) and 6-month-old (*n* = 2) GCLC-KO mice, representing transcriptomic heterogeneity within the microglial population. **D** The violin plot showing the log2 expression of microglia markers in microglial subclusters extracted from the snRNA-seq dataset. **E** A pathway enrichment analysis of genes that were specifically expressed in microglial subcluster 2 and detected in 3-month-old GCLC-KO mice was performed. The analysis was conducted using Enrichr (https://maayanlab.cloud/Enrichr/), utilizing the Reactome Pathways 2024 database. The terms represented enriched pathways. The overlap shows the ratio of input genes included in each pathway. The adjusted *P*-values indicated statistically significant difference after correction for multiple testing. The odds ratios reflect the strength of the association between the input genes and each pathway. The combined scores integrate the *P*-value and z-score to rank pathway enrichment. Supplemental Data 3 shows the complete list of pathways. **F** Dot plot showing the expression levels of the *Plau*, *Plaur*, and DAM markers across the microglial subclusters derived from GCLC-KO mice. Each dot represents the average expression of a gene in a given cluster, with dot size indicating the percentage of expressing cells and color intensity reflecting average expression levels

Considering that rod-shaped microglia express DAM markers, the molecular mechanisms underlying their unique morphology and potential functional roles were investigated. To this end, snRNA-seq was performed using cortical tissues collected from 3- and 6-month-old GCLC-KO mice. At 3 months, rod-shaped microglia were abundant, but DAM marker–positive microglia were relatively rare, making it difficult to define transcriptomic clusters based on this time point alone. In contrast, 6-month-old samples contained a larger population of amoeboid, DAM-like microglia, allowing for more robust clustering. We therefore integrated the two datasets, used the 6-month data to define DAM-like clusters, and subsequently examined gene expression in 3-month-derived cells within those clusters to identify factors enriched during the rod-shaped stage. Single nuclei were isolated from the cortical region, where rod-shaped microglia are more frequently observed (Fig. 5A). Library preparation and sequencing were conducted using the Chromium platform (10X Genomics). Quality control was performed to ensure high-quality data for clustering and downstream analyses (Supplemental Fig. 4A). UMAP visualization revealed distinct clusters of all cell-derived nuclei (Fig. 5B). Cell type annotation was conducted based on the expression of canonical marker genes, and cluster 5 was identified as the microglial population due to its high expression of *Hexb* (Supplemental Fig. 4B).

Subclustering of cluster 5 (microglia) was performed. Figure 5C shows the resulting UMAP projection, and Supplemental Fig. 5A depicts the UMAP plots for individual samples. To further characterize each microglial subcluster, the expression of microglial marker genes in violin plots was examined (Fig. 5D). Subcluster 2 exhibited an elevated expression of *Hif1a*, a DAM-associated marker, thereby indicating a DAM-like phenotype. In contrast, subcluster 4 showed a high expression of *Ifit3*, which suggested that this cluster corresponds to the IRM. There were some variations in the abundance of homeostatic microglial clusters between samples. However, a DAM-like subcluster (subcluster 2) was consistently present across all samples. Supplemental Fig. 5B depicts a heatmap of the characteristic genes for each microglial subcluster. The rod-shaped microglia expressed DAM markers (Fig. 4), which indicated that they share transcriptional characteristics with the DAM-like Subcluster 2, rather than forming a distinct microglial cluster. Given that rod-shaped microglia were most frequently observed at 3 months of age and they exhibited a DAM-like gene expression pattern, we focused on identifying genes specifically expressed in DAM in 3-month-old GCLC-KO mice. According to these data, we extracted genes that were strongly expressed both in the overall DAM-like subcluster 2 and the samples collected from the 3-month-old GCLC-KO mice. Supplemental Data 2 shows these genes.

To explore the biological pathways associated with these genes, a pathway enrichment analysis was performed using Enrichr (https://maayanlab.cloud/Enrichr/) with the Reactome 2024 database. Figure 5E shows the pathways with an adjusted *p*-value of < 0.05, and Supplemental Data 3 depicts the full list of enriched pathways. Based on the result of the pathway enrichment analysis, the dissolution of fibrin clot pathway was identified as the most significantly enriched pathway in the DAM-like subcluster 2 of the 3-month-old GCLC-KO mice. This pathway plays an essential role in extracellular matrix (ECM) remodeling by mediating the degradation of fibrin, a key component of the ECM. In the central nervous system, the ECM acts as a structural barrier that regulates microglial migration and morphological changes [28, 29]. Therefore, an effective ECM remodeling is essential for microglia to extend their processes and migrate along neuronal fibers. In particular, the formation of rod-shaped microglia is characterized by their alignment along neuronal fibers and the elongation of cellular processes, which are likely to be closely associated with ECM remodeling. Within the dissolution of fibrin clot pathway, urokinase-type plasminogen activator (uPA, encoded by *Plau*) and the uPA receptor (*Plaur*) play central roles. uPA promotes the conversion of plasminogen to plasmin, leading to fibrin and ECM degradation. Taken together, these findings indicate that *Plau* (uPA) is likely to play an important role in the formation of rod-shaped microglia, potentially through its involvement in ECM remodeling and intracellular signaling. Although *Plaur* was also upregulated, we selected *Plau* for follow-up due to its more restricted expression pattern within the DAM-like cluster. Therefore, we focused our subsequent analysis on *Plau* to further investigate its possible role in rod-shaped microglia.

### Rod-shaped microglia exhibited uPA/GAP43 signaling activation

To assess whether the *Plau* expression is a common feature of DAM, publicly available single-cell RNA-seq datasets from two AD mouse models (5XFAD [GSE140510] and *App*^NL−G−F^ KI mice [GSE127893]) were first analyzed. In both models, the *Plau* expression was enriched in DAM subclusters, similar to the canonical DAM markers such as *Itgax* and *Cst7* (Supplemental Figs. 6A, 6 C). In the 5XFAD model, TREM2 plays an essential role in the transition from stage 1 to stage 2 DAM, which is characterized by the upregulation of genes such as *Itgax* and *Cst7*. Consistent with this finding, the *Plau* expression in TREM2-KO X 5XFAD mice was significantly reduced compared with that in 5XFAD mice (Supplemental Fig. 6B). This suggests that *Plau* is part of the TREM2-dependent transcriptional program associated with stage 2 DAM formation. Similarly, in *App*^NL−G−F^ mice, the *Plau* expression progressively increased with age, which is correlated with disease progression and DAM activation (Supplemental Fig. 6D). Collectively, these results show that *Plau* is consistently upregulated in DAM across different AD mouse models and may play a key role in TREM2-mediated microglial activation and potentially in morphological transitions during neurodegeneration.

**Fig. 6:**
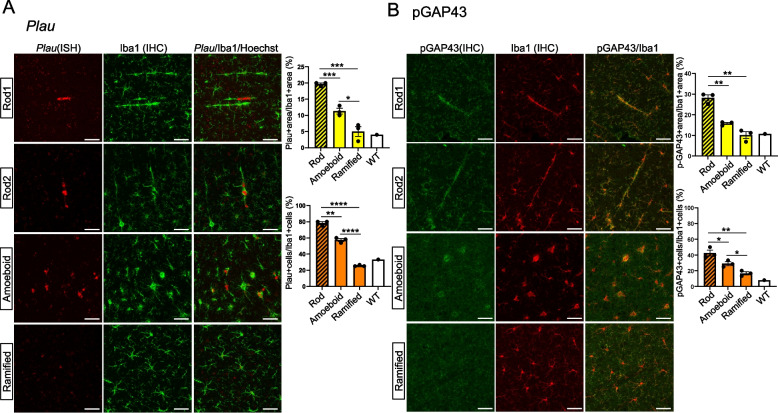
Rod-shaped microglia expressed *Plau* and phosphorylated GAP43. **A** The brain sections from 3-month-old GCLC-KO mice were subjected to dual staining using in situ hybridization with RNAscope (*Plau*) and immunostaining with the Iba1 antibody. Each panel depicts regions with abundant rod, amoeboid, and ramified microglia (Supplemental Fig. 3A). The scale bar represents 50 μm. The upper graph indicates the ratio of the RNAscope-positive area to the Iba1-positive area. Meanwhile, the lower graph represents the ratio of RNAscope-positive cells to Iba1-positive cells. The values related to the rod, amoeboid, and ramified microglia in GCLC-KO mice were expressed as the mean relative levels ± standard error of the mean (*n* = 3). The reference values for the microglia in wild-type mice (white bar) are also provided. Supplemental Fig. 7A shows the staining images of wild-type mice. **B** The upper graph represents the ratio of the co-localized pGAP43/Iba1-positive area to the total Iba1-positive area (yellow bars), and the lower graph represents the ratio of pGAP43-positive cells to Iba1-positive cells (orange bars). The scale bar indicates 50 μm. The values related to the rod, amoeboid, and ramified microglia in GCLC-KO mice were expressed as the mean relative levels ± standard error of the mean (*n* = 3). The reference values for the microglia in wild-type mice (white bar) were also provided. Supplemental Fig. 6B shows the staining images of the wild-type mice. *p < 0.05, **p < 0.01, *** p < 0.001, **** p < 0.0001

Next, based on these findings, the *Plau* expression in rod-shaped microglia was investigated. Notably, *Plau* was predominantly expressed in a subset of rod-shaped microglia in GCLC-KO mice (Fig. 6A, Supplemental Fig. 7A). Considering that uPA facilitates cell migration via ECM remodeling, this finding suggests that rod-shaped microglia may exhibit enhanced migratory capacity and morphological plasticity. In addition to ECM remodeling, uPA signaling activates GAP43, a key molecule in axonal growth and regeneration in neurons [30]. To explore whether GAP43 is also involved in microglial elongation, the expression of phosphorylated GAP43 (pT172-GAP43) in GCLC-KO mice (Fig. 6B, Supplemental Figs. 7B, 7 C) was examined. The GAP43 function is regulated by its phosphorylation at T172, which is associated with axonal growth activity [31, 32 ]. Strong pT172-GAP43 signals were observed in a subset of rod-shaped microglia, and these signals appeared to be more prominent than those observed in amoeboid microglia. Taken together, these findings raise the possibility that uPA signaling and GAP43 activation contribute to the morphological characteristics of rod-shaped microglia.

## Discussion

This study showed that rod-shaped microglia form in response to oxidative stress caused by glutathione deficiency. These microglia did not exhibit a uniform gene expression pattern. However, they presented with an activation state similar to that of DAM. Moreover, uPA signaling and pGAP43 were considered as potential contributors to their formation, thereby indicating that extracellular matrix remodeling and intracellular signaling play a role in shaping their morphology. Rod-shaped microglia have been observed in postmortem brains across a wide range of conditions, including aging, and neurodegenerative diseases, such as AD [33, 34]. However, their functional significance and formation mechanisms remain largely unknown. Considering their presence in ischemic rat brain slices [20], it is plausible that reactive oxygen species contribute to their formation. Given the presence of oxidative stress and early neuronal abnormalities in the cortex of GCLC-KO mice, it is conceivable that rod-shaped microglia may arise as part of an adaptive response to neuronal distress.

Recent studies have shown that DAM and related subpopulations arise under pathological conditions. However, their exact cellular characteristics remain unclear. Our study provided evidence showing that DAM could adopt not only the well-recognized amoeboid morphology but also the rod-shaped form. This observation expands the current understanding on DAM heterogeneity. While our classification is based on well-defined morphological criteria, we acknowledge that this approach alone may not fully capture the molecular heterogeneity of microglial subtypes. Therefore, we performed transcriptomic profiling using snRNA-seq to complement morphology-based classification and to explore potential transitional relationships between rod-shaped and amoeboid microglia. Some variation in microglial subset distribution was observed between 3-month-old samples; however, key DAM-like features, including those in Cluster 2, were consistently detected. Publicly available wild-type data (Supplementary Fig. 6) also show low expression of DAM-associated genes, supporting the disease relevance of the transcriptomic changes observed in GCLC-KO mice. Although rod-shaped microglia shared several DAM-associated gene expression features, it remains unclear whether they represent a transcriptionally distinct microglial subpopulation. A comprehensive genome-wide comparison will be necessary to clarify this point.

Interestingly, Lana et al. [20] reported that rod-shaped microglia appear early after ischemic injury and subsequently transition into amoeboid microglia. Hence, rod-shaped microglia may represent an intermediate activation state, preceding the fully phagocytic amoeboid phenotype as neurodegenerative progresses. Nonetheless, the reason why DAM-like microglia occasionally adopt a rod-shaped morphology remains unclear. This unique morphology can facilitate specific interactions with neuronal processes, influences microglial migration, or modulates neuroinflammatory responses in a distinct manner. To further explore these possibilities, advanced spatial transcriptomics technologies could be utilized. Recent advancements in spatial resolution now allow transcriptomic profiling at the subcellular levels [35–37], thereby offering an opportunity to investigate morphology-specific gene expression patterns and microglial interactions with surrounding cells within the central nervous system.

A recent study [38] (*Preprint*) identified rod-shaped microglia in the brain and spinal cord of a TDP-43-related amyotrophic lateral sclerosis mouse model. Similar to our findings, these microglia were aligned along neuronal processes, and they exhibited DAM-like activation. This suggests that rod-shaped microglia are not exclusive to oxidative stress-induced neurodegeneration. In fact, they may also arise in neurodegenerative disorders associated with proteinopathy and abnormal neuronal activity [38]. Rod-shaped microglia have been observed aligning along injured axons and are hypothesized to play a role in neuroprotection by facilitating the reorganization of neuronal circuitry following CNS injuries [17, 21]. In GCLC-KO mice, these cells may arise in response to early neuronal alterations, potentially reflecting a unique morphological response to neuronal stress. Thus, they may act as early responders to neuronal distress signals.

Our study is based on the notion that *Plau* (encoding uPA) is upregulated in rod-shaped microglia, thereby indicating its potential role in maintaining their morphology. Tam et al. [21] showed that primary microglia cultured on laminin-coated dishes formed rod-like structures along scratch lines. Moreover, this elongated shape was maintained. uPA is well known for its ability to degrade the ECM and facilitate cell migration, particularly in cancer cells. The uPA receptor, which is its receptor, is expressed in the microglia and is upregulated during neuroinflammation. Moreover, uPA induces the phosphorylation of GAP43, thereby promoting synaptic remodeling via NMDA receptor activation in neurons. Based on our findings, uPA may similarly regulate microglial morphology via GAP43 activation in rod-shaped microglia [30]. However, the precise mechanisms by which uPA signaling promotes GAP43 phosphorylation in the microglia remain unclear, and it should be further investigated. In addition, *Plaur* was also upregulated in the DAM-like cluster. While we focused on Plau due to its more restricted expression, the co-expression of *Plau* and *Plaur* suggests that uPA–uPAR signaling may play a role in shaping rod-shaped microglia, warranting future functional studies.

GCLC-KO mice exhibit diverse microglial morphological changes, in addition to neuronal loss. Therefore, they can be a valuable model for studying microglial plasticity in response to oxidative stress and neurodegeneration. Although GCLC-KO mice do not model glutathione synthetase deficiency per se—which is caused by mutations in the GSS gene and presents with severe systemic glutathione depletion and neurological symptoms—they reproduce key features of brain redox imbalance. This makes them useful for investigating oxidative stress-induced neuropathology. In addition, several studies have reported that glutathione levels are decreased in the brains of individuals with mild cognitive impairment (MCI) and AD, suggesting that redox imbalance is a relevant feature of AD pathophysiology [39, 40] Cecchini et al., 1999). Consistent with these human findings, we previously showed that GCLC-KO mice exhibit an increased Aβ42/Aβ40 ratio and enhanced tau aggregation [41], both of which are key features of AD. Although this model does not fully recapitulate all aspects of AD, it represents a useful system for studying oxidative stress–driven amyloid and tau pathologies. These findings, together with the emerging evidence of redox imbalance in AD, support the utility of GCLC-KO mice as a model to study the pathophysiological relevance of rod-shaped microglia.

Considering the widespread presence of rod-shaped microglia in the postmortem brains of aging individuals and those with ischemia, dementia with Lewy bodies, and AD, understanding their role in disease progression could provide novel insights into microglial function. Regulating microglial responses, including those of rod-shaped microglia, may have implications on the progression of neurodegenerative diseases. Whether rod-shaped microglia exacerbate or inhibit neuronal damage remains unclear. However, our study underscores the importance of investigating how microglial morphology influences neuroinflammatory responses and neurodegeneration. Nevertheless, further research using advanced transcriptomics and functional assays must be conducted to completely elucidate their role in central nervous system pathologies.

## Supplementary Information

Supplementary Materials 1: Supplementary Figures S1–S7. Additional imaging data, quantification analyses, and validation experiments. Supplementary Tables S1–S2: Lists of antibodies and probes used for RNAscope.

Supplementary Materials 2: Supplementary Table S3. Detailed information on animals and methods used for image-based and Western blot quantification.

Supplementary Materials 3: Supplementary Data 1. Three-dimensional reconstruction movie of rod-shaped microglia observed in cleared brain tissues collected from GCLC-KO mice (related to Fig. 2A).

Supplementary Materials 4: Supplementary Data 2. List of genes specifically expressed in the DAM-like clusters of 3-month-old GCLC-KO mice.

Supplementary Materials 4: Supplementary Data 3. Complete results of the pathway enrichment analysis performed using the Reactome 2024 database.

Supplementary Materials 5: Supplementary Data 4. Statistical Analysis of DAM-Associated Genes in 5XFAD and *App*^NL-G-F^ scRNA-seq Datasets.

## Data Availability

The single-nucleus RNA sequencing (snRNA-seq) datasets generated during the current study have been deposited in the GEO database under accession number GSE301920 (BioProject: PRJNA1286132) and will be publicly available upon publication. The public scRNA-seq datasets analyzed in this study are available from the GEO database under accession numbers GSE140510 and GSE127893.

## Abbreviations

AD: Alzheimer’s disease
ECM: Extracellular matrix
DAM: Disease-associated microglia
ARM: Activated response microglia
IRM: Interferon response microglia
Plau: Urokinase-type plasminogen activator gene
uPA: Urokinase-type plasminogen activator
GAP43: Growth-associated protein 43
GCLC: Glutamyl cysteine ligase catalyic subunit
RT-PCR: Reverse transcription polymerase chain reaction
scRNA-seq: Single-cell RNA sequencing
snRNA-seq: Single-nucleus RNA sequencing
