# Supplementary material for: Rod-shaped microglia represent a morphologically distinct subpopulation of disease-associated microglia
Source: J Neuroinflammation. 2025 Jul 16;22:184. doi: 10.1186/s12974-025-03504-5 (PMC12269120; doi:10.1186/s12974-025-03504-5)
Supplement: Supplementary file 1 — Supplementary Materials 1: Supplementary Figures S1–S7. Additional imaging data, quantification analyses, and validation experiments. Supplementary Tables S1–S2: Lists of antibodies and probes used for RNAscope. [file 12974_2025_3504_MOESM1_ESM.pdf]

Supplemental Figure 1

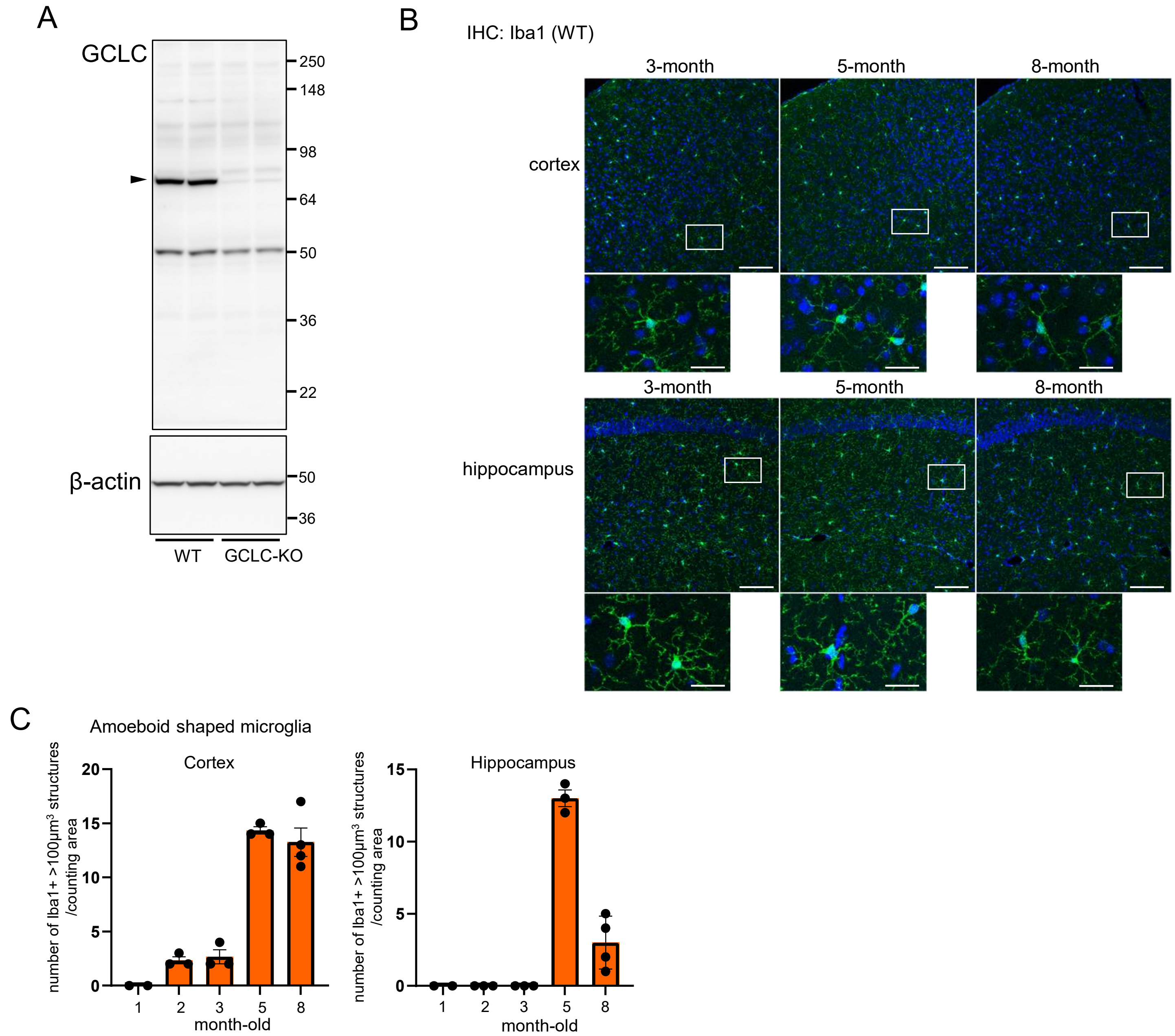

Supplemental Figure 1 Immunostaining and quantification of Iba1-positive microglia in wild-type and GCLC-KO mice
(A)Western blot analysis of GCLC protein expression in cortical lysates from wild-type (WT) and GCLC knockout (KO) mice. A band corresponding to GCLC (~73 kDa, indicated by arrow) is clearly detected in WT mice and reduced in GCLC-KO mice, confirming efficient gene deletion at the protein level. β-actin was used as a loading control.(B) Brain sections from 3- to 8-month-old wild-type mice were immunostained with the Iba1 antibody. Lower panels each display magnified views of the boxed areas in the corresponding upper panels, highlighting microglial morphology in greater detail. The scale bar represent 100 μm (upper panels) and 25 μm (lower panels). (C) The number of Iba1-positive structures with a soma area >100 μm² (defined as amoeboid-shaped microglia) was counted within the x10 confocal image fields of GCLC-KO mouse brain sections shown in Fig. 1A and 1B. Values from 2 to 8 months are expressed as the mean relative levels ± SEM (n=3 or 4 mice).

Supplemental Figure 2

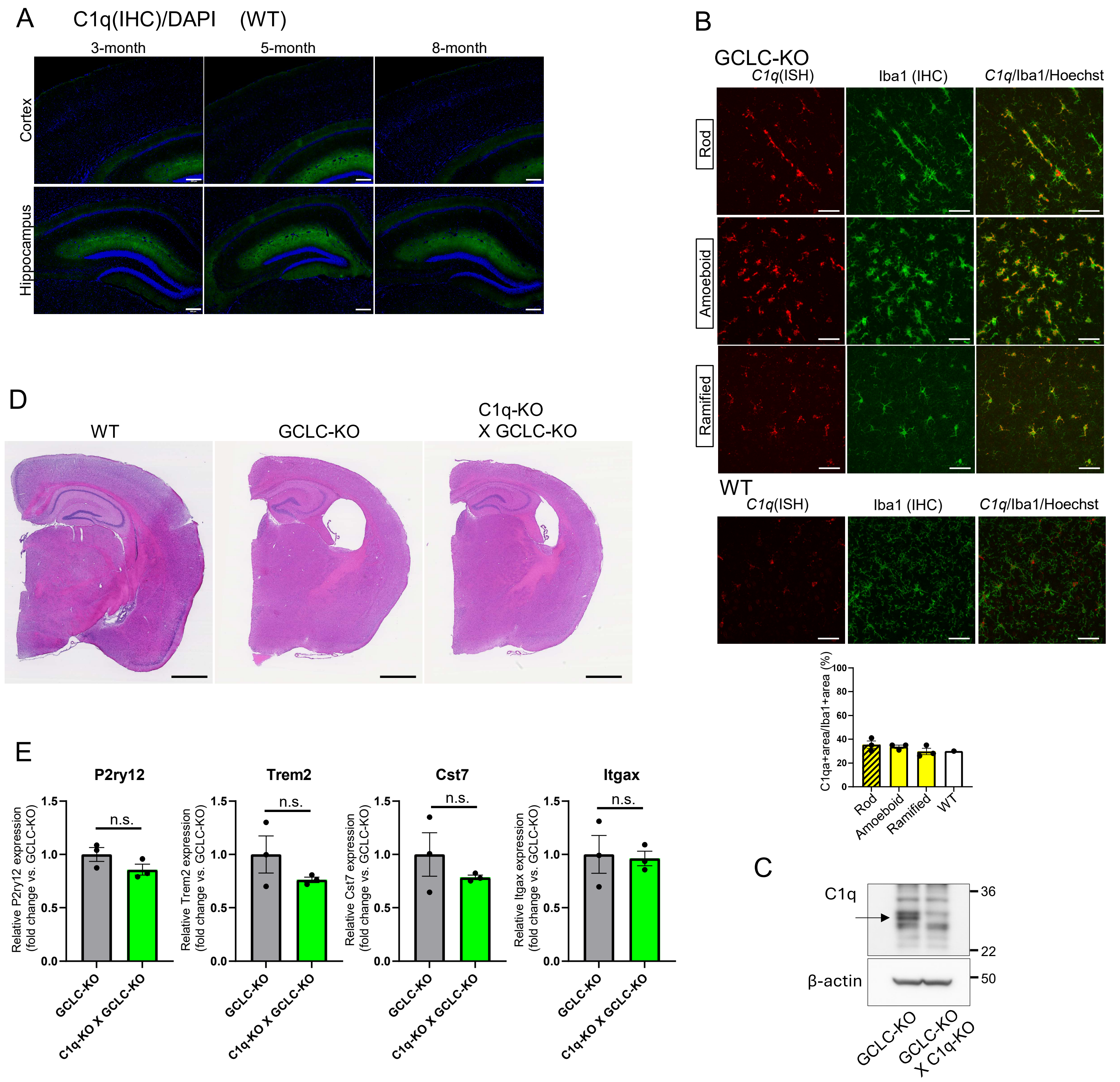

Supplemental Figure 2 Microglial morphology in wild-type, GCLC-KO, and C1q-KO × GCLC-KO mice
(A) Brain sections from 3- to 8-month-old wild-type mice were immunostained with the C1q antibody. Upper panels show cortical region and lower panels show hippocampal region. The scale bar represents 200 μm. (B) The brain sections from 3-month-old GCLC-KO mice were subjected to dual staining using in situ hybridization with RNAscope (*C1qa*) and immunostaining with the Iba1 antibody. Each panel depicts regions with abundant rod, amoeboid, and ramified microglia (refer to Supplemental Figure 3A). The scale bar represents 50 μm. The graph represents the ratio of RNAscope-positive area to Iba1-positive area. (C) Western blot analysis of C1q protein expression in cortical lysates from GCLC-KO and GCLC-KO X C1q-KO. The arrow indicates the C1q band. C1q was detected in GCLC-KO mice but was absent in GCLC-KO X C1q-KO mouse, confirming successful knockout. (D) Brain sections from 5-month-old wild-type mice, 9-month-old GCLC-KO mice, and 9-month-old C1q-KO X GCLC-KO mice were stained with hematoxylin and eosin (H&E). Scale bar represents 1 mm. (E) Relative mRNA expression levels of P2ry12, Trem2, Cst7, and Itgax were measured in the cerebral cortex of GCLC-KO mice and C1q-KO X GCLC-KO double mutant mice at 3 months of age. Gene expression levels were normalized to GAPDH, and values are shown relative to the GCLC-KO group, which was set to 1. Data are presented as mean ± SEM (n = 3 per group). Statistical significance was assessed using Student's t-test. n.s., not significant

Supplemental Figure 3

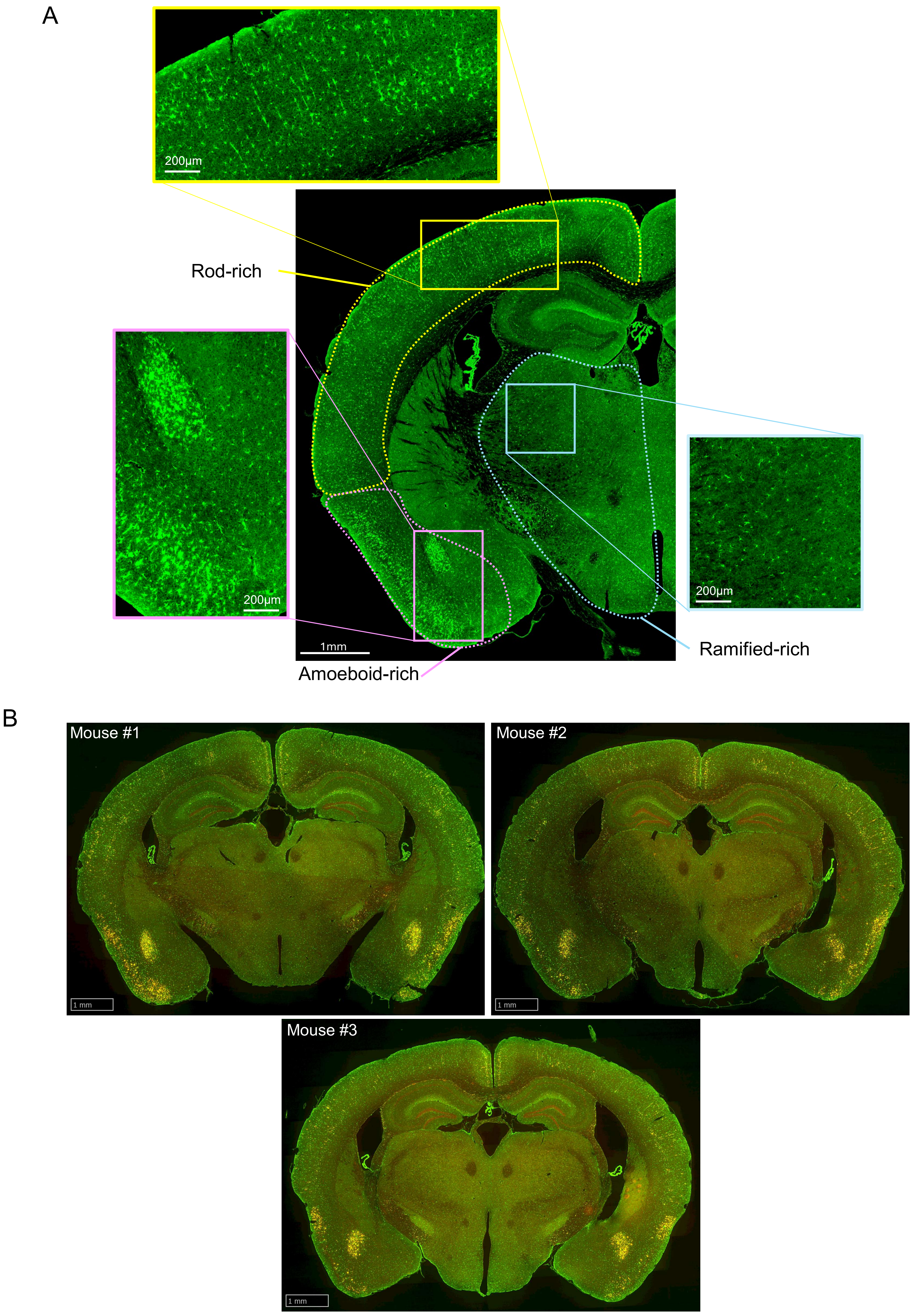

Supplemental Figure 3 (continued)

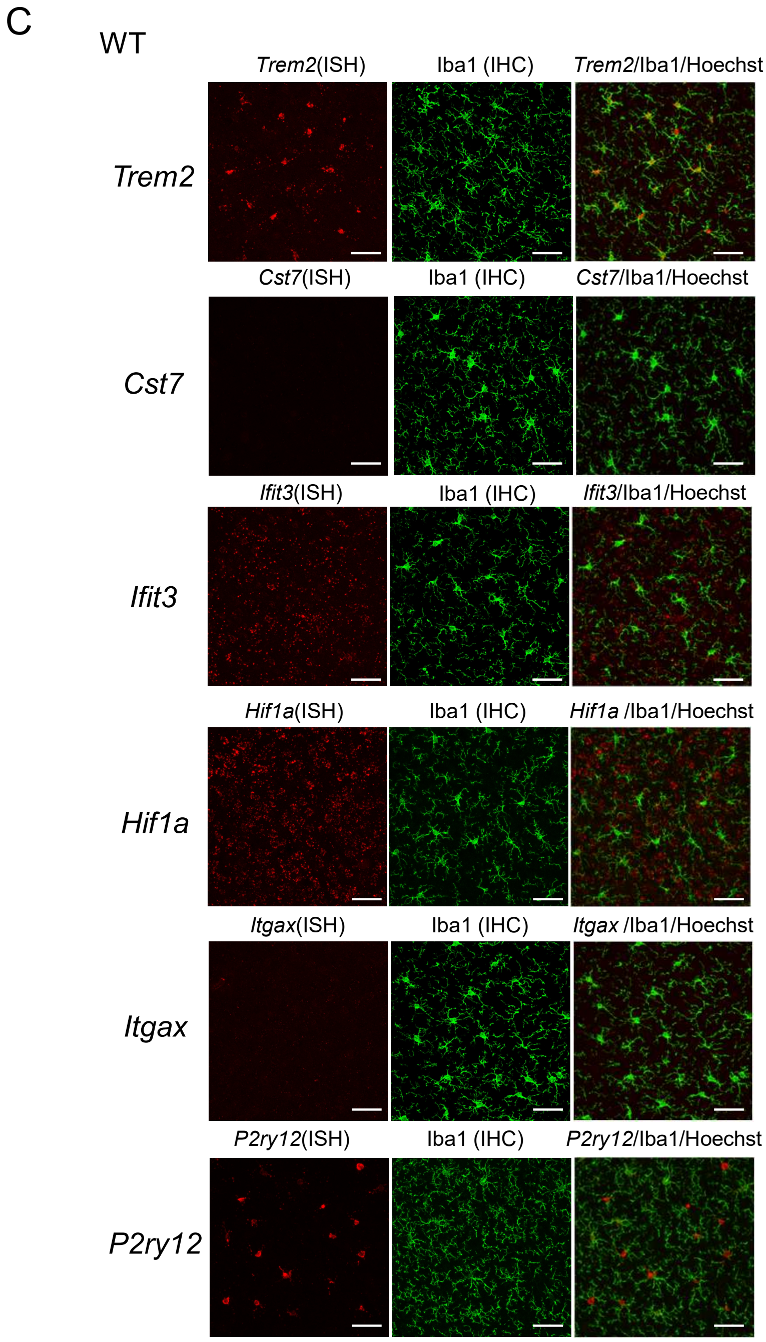

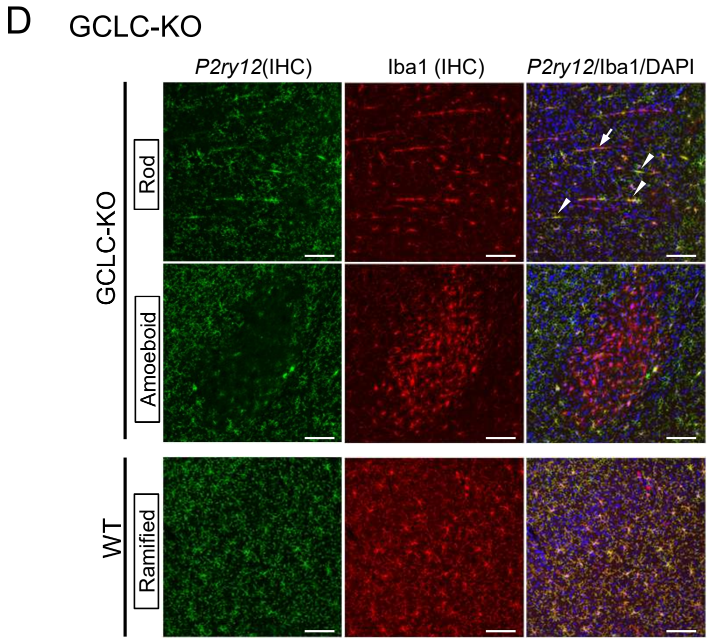

Supplemental Figure 3 Microglial morphology and P2ry12 expression in WT and GCLC-KO mice

(A) Brain sections from 3-month-old GCLC-KO mice were immunostained with the Iba1 antibody. Regions enriched in rod-shaped, amoeboid, and ramified microglia are outlined with dashed lines. Areas enclosed by solid squares indicate regions shown at higher magnification. In situ hybridization (ISH) analysis was performed on representative locations within each morphologically enriched region. (B) Whole-brain sections from three 3-month-old GCLC-KO mice were stained with an anti-Iba1 antibody (immunohistochemistry) and Cst7 probes (RNAscope). Morphology-enriched regions (rod-, amoeboid-, and ramified-type microglia) were manually annotated and used for quantification shown in Figure 4. The same staining and image acquisition protocol was applied to other analyzed genes (not shown). (C)The brain sections from 3-month-old WT mice were subjected to dual staining using in situ hybridization with RNAscope and immunostaining with the Iba1 antibody. Each panel depicts regions with abundant rod, amoeboid, and ramified microglia. The scale bar represents 50 µm.(D) Brain sections from 3-month-old WT and GCLC-KO were immunostained with the P2ry12 and Iba1 antibody. The scale bar represents 50 µm.

Supplemental Figure 4

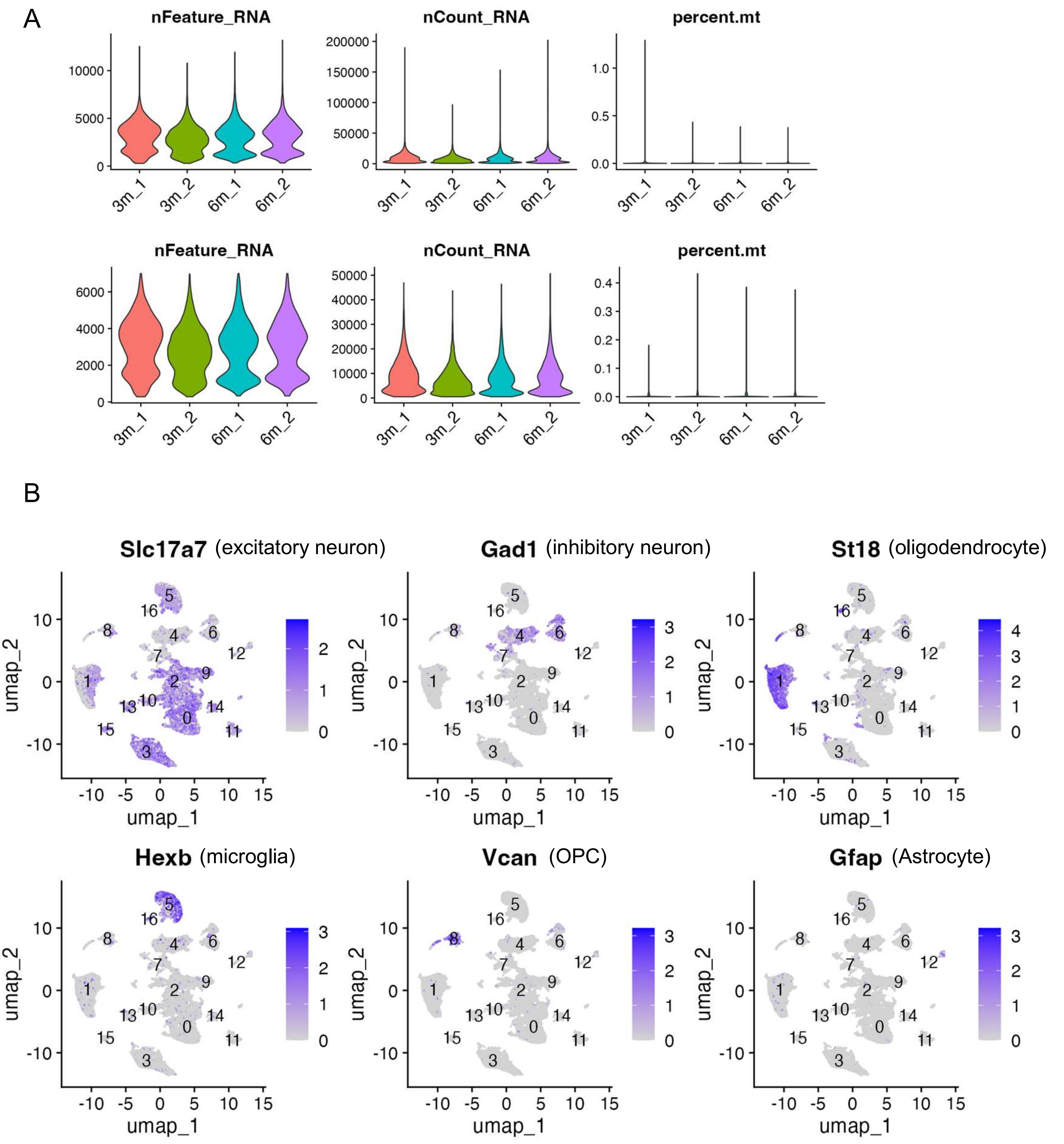

Supplemental Figure 4. Quality control and cell type annotation in microglial clustering analysis

(A) Violin plots showing the distributions of nFeature_RNA, nCount_RNA, and percent.mt for each sample. Data before filtering (top) and after filtering (bottom) are presented. Nuclei with more than 7,000 detected genes or with mitochondrial gene content greater than 1% were excluded during quality control. Samples labeled 6m_1 and 6m_2 represent 6-month-old mice, while 3m_1 and 3m_2 represent 3-month-old mice. (B) Feature plots showing the expression patterns of representative cell type-specific markers across all clusters: Slc17a7 (excitatory neurons), Gad1 (inhibitory neurons), Atp1a2 (oligodendrocytes), Hexb (microglia), Vcan (oligodendrocyte precursor cells; OPCs), and Gfap (astrocytes). Color intensity indicates relative expression levels (log-normalized).

Supplemental Figure 5

A

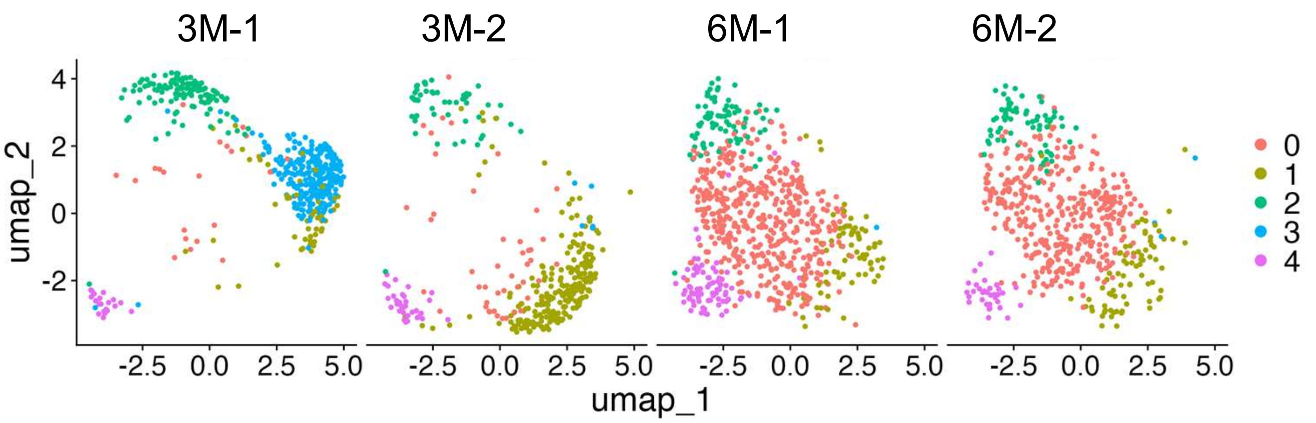

B

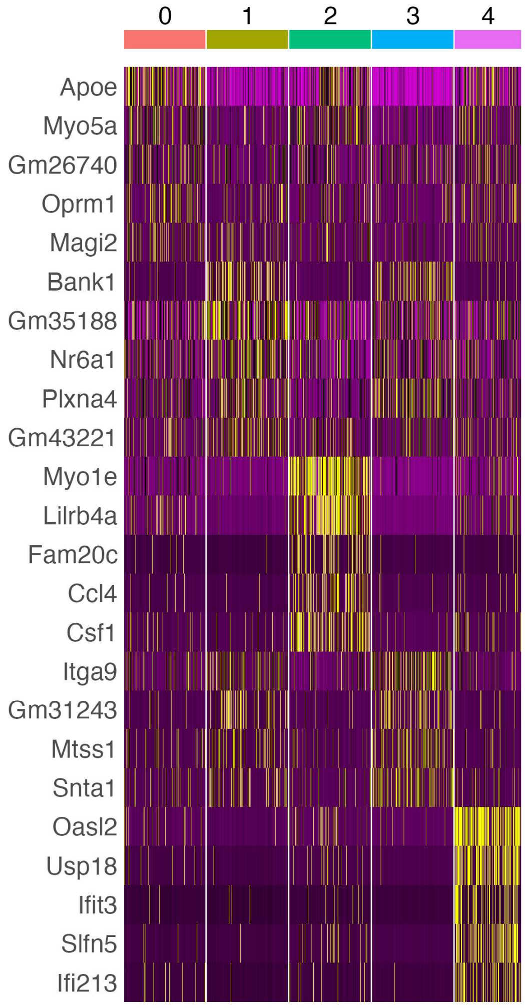

Supplemental Figure 5 UMAP visualization of microglial subclusters

(A) UMAP plots showing the subclustering of microglia from the snRNA-seq dataset, displayed separately for each sample. Rather than using an integrated dataset, microglial subclusters are visualized for individual samples: 3M-1 (3-month-old #1), 3M-2 (3-month-old #2), 6M-1 (6-month-old #1), and 6M-2 (6-month-old #2). (B) Heatmap illustrating the expression profiles of key marker genes across microglial subclusters identified in the single-nucleus RNA-seq dataset. Columns correspond to individual subclusters, and rows represent marker genes selectively enriched in each cluster. Gene expression levels are scaled and color-coded, with red indicating higher expression and blue indicating lower expression.

Supplemental Figure 6

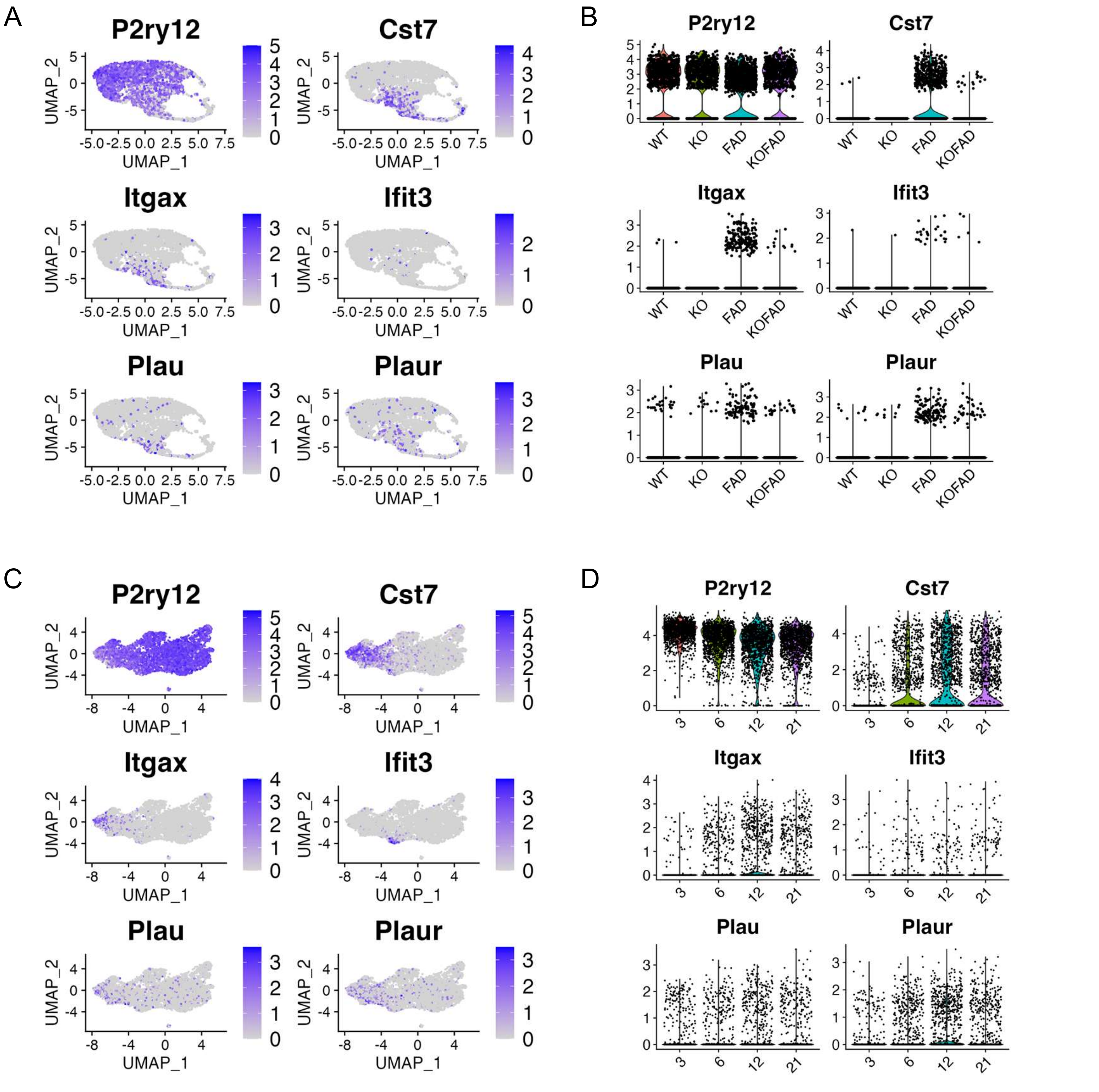

Supplemental Figure 6. **Comparative analysis of Plau and DAM marker expression in Alzheimer's disease mouse models**

(A, C) FeaturePlot visualizations showing the expression of Plau and microglial markers in microglial clusters from public scRNA-seq datasets of 5XFAD (A) and *App*$^{NL\text{-}G\text{-}F}$ KI (C) mice. Plau expression is enriched in the DAM cluster in both models, similar to canonical DAM markers such as Itgax and Cst7. (B) Plau expression in microglia from WT, TREM2-KO, 5XFAD, and TREM2-KO X 5XFAD mice.(D) Plau expression in microglia from *App*$^{NL\text{-}G\text{-}F}$ KI mice at 3, 6, 12, and 21 months of age. These results support the notion that Plau expression in DAM is a common feature across different Alzheimer's disease mouse models.

Supplemental Figure 7

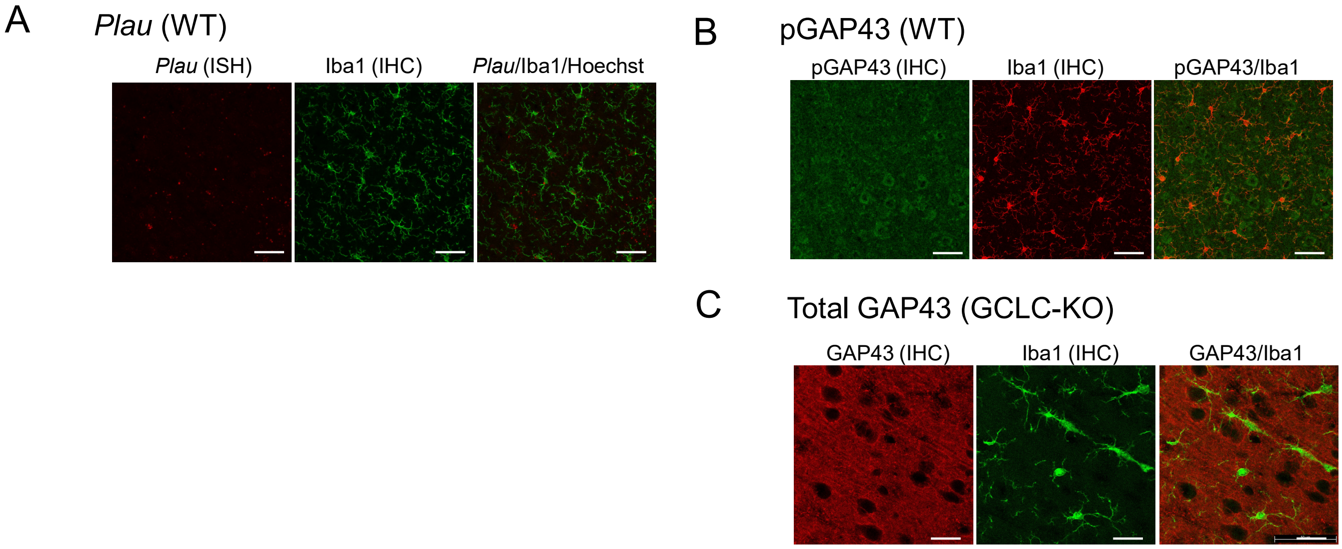

Supplemental Figure 7 Expression pattern of *Plau* and GAP43

(A) Brain sections from 3-month-old WT mice were subjected to dual staining using in situ hybridization with RNAscope (*Plau*) and immunostaining with the Iba1 antibody. The scale bar represents 50μm (right panels).(B, C) Brain sections from 3-month-old WT (B) or GCLC-KO (C) were immunostained with the phosphorylated GAP43 (B) or total GAP43 (C) antibody. The scale bar represents 50μm.

Supplemental Table 1. Antibodies used for the immunohistochemistry (IHC) and western blotting analysis.

The following antibodies were used at the indicated dilutions. For antibodies used in IHC, the lot numbers are provided, except for the anti-MBP antibody, for which the lot number is unavailable.

Proteins	Product Number (Lot number)	Dilution IHC	WB
Iba1	Fujifilm #013-27691 (#SKG4637)	1:200	
	abcam #ab5076 (#GR3178800-2)	1:200	
C1q	abcam #ab1824541 (#GR290355-11)	1:500	1:1000
MAP2	Leinco Technologies #M121 (unavailable)	1:50	
MBP	R&D Systems #MAB42282 (#CJLN029091)	1:1000	
Olig2	abcam #ab109186 (#GR210294-20)	1:200	
CD31	BioLegend #102501 (#B295545)	1:50	
P2ry12	Produced by Sahara	1:5000	
GAP43(total)	Nittobo Medical #MSFR101670	1:200	
p-GAP43	Fujifilm #017-25411	1:100000 (TSA)	
GSDMD	abcam #ab219800		1:2000
GSDME	abcam #ab215191		1:5000
GCLC	ATLAS ANTIBODIES #HPA036359		1:5000
β-actin	Sigma #A5441		1:5000

Supplemental Table 2. Probes for RNAscope

The probes used in the RNAscope analysis are purchased from Advanced Cell Diagnostics. These probes recognize the mouse sequences for each gene. The product numbers were as follows.

Genes	Product Number
Trem2	#404111
Hif1a	#313821
Cst7	#498711
Itgax	#311501
Ifit3	#508251
P2ry12	#317601
C1qa	#441221
Plau	#433641
